# A bird’s eye view: using circuit theory to study urban landscape connectivity for birds

**DOI:** 10.1007/s10980-017-0548-1

**Published:** 2017-06-28

**Authors:** Darren R. Grafius, Ron Corstanje, Gavin M. Siriwardena, Kate E. Plummer, Jim A. Harris

**Affiliations:** 1 School of Water, Energy and Environment, Cranfield University, Cranfield, Bedfordshire MK43 0AL UK; 2 British Trust for Ornithology, The Nunnery, Thetford, Norfolk IP24 2PU UK; 3 Centre for Ecology and Conservation, College of Life and Environmental Sciences, University of Exeter, Penryn Campus, Cornwall, TR10 9FE UK

**Keywords:** Circuitscape, Connectivity, Circuit theory, Urban, *Parus major*, *Cyanistes caeruleus*, Landscape structure, Ecosystem service, Modelling

## Abstract

**Context:**

Connectivity is fundamental to understanding how landscape form influences ecological function. However, uncertainties persist due to the difficulty and expense of gathering empirical data to drive or to validate connectivity models, especially in urban areas, where relationships are multifaceted and the habitat matrix cannot be considered to be binary.

**Objectives:**

This research used circuit theory to model urban bird flows (i.e. ‘current’), and compared results to observed abundance. The aims were to explore the ability of this approach to predict wildlife flows and to test relationships between modelled connectivity and variation in abundance.

**Methods:**

Circuitscape was used to model functional connectivity in Bedford, Luton/Dunstable, and Milton Keynes, UK, for great tits (*Parus major*) and blue tits (*Cyanistes caeruleus*), drawing parameters from published studies of woodland bird flows in urban environments. Model performance was then tested against observed abundance data.

**Results:**

Modelled current showed a weak yet positive agreement with combined abundance for *P. major* and *C. caeruleus*. Weaker correlations were found for other woodland species, suggesting the approach may be expandable if re-parameterised.

**Conclusions:**

Trees provide suitable habitat for urban woodland bird species, but their location in large, contiguous patches and corridors along barriers also facilitates connectivity networks throughout the urban matrix. Urban connectivity studies are well-served by the advantages of circuit theory approaches, and benefit from the empirical study of wildlife flows in these landscapes to parameterise this type of modelling more explicitly. Such results can prove informative and beneficial in designing urban green space and new developments.

## Introduction

Urban landscapes with high functional connectivity and native vegetation biodiversity are associated with increased abundance and stability of bird populations (Rosenfeld 2012). Human interactions with birds have in turn been identified as one of the most readily recognised wildlife interactions that most urban residents experience regularly, and have been linked with benefits to psychological well-being and a sense of connectedness to nature (Fuller et al. 2007; Jones and Reynolds 2008; Jones 2011; Luck et al. 2011; Dallimer et al. 2012; Galbraith et al. 2014; Belaire et al. 2015; Cox and Gaston 2016). Songbirds, in particular, are viewed favourably by urban residents, tending to be unobtrusive, brightly coloured, and rarely a source of human–avian conflict, while exhibiting behaviours that people often find interesting to watch (Cox and Gaston 2015). Better understanding of the movement of birds in urban landscapes, and how these movements may be influenced by the structure of the landscape, can also be used to inform urban planning and design for biodiversity and sustainability goals. Such an understanding therefore has the potential to benefit not only human well-being, but bird conservation as well.

Landscape connectivity is fundamental to linking ecological function to landscape form. It describes the degree to which an environment’s spatial configuration facilitates biological flows, often in the context of organisms travelling within and between habitat patches (Tischendorf and Fahrig 2000). Structural connectivity pertains to the underlying landscape geometry, such as corridor width and distance between patches, whereas functional connectivity seeks to consider the specific needs and behaviours of a target species or species group (Uezu et al. 2005). Landscape structure is relatively straightforward to quantify through the calculation of various landscape pattern metrics, such as are made available by the software program Fragstats (McGarigal et al. 2012), and as such has been widely studied across diverse ecological systems. However, uncertainty persists concerning the interpretation of many such metrics and their effectiveness as indicators of ecological flows, functions and health (Pascual-Hortal and Saura 2006; Baguette and Van Dyck 2007; Kupfer 2012). Studies of structural connectivity in urban settings have highlighted its importance and utility as an aid to planning (e.g. Marulli and Mallarach 2005; Yu et al. 2015); however, the unique complexity and high heterogeneity of these environments renders them particularly difficult to study in this regard, and numerous gaps remain in our understanding of how urban form influences ecological function (LaPoint et al. 2015).

In order to consider connectivity in a functional sense, methods are needed that can model the flow dynamics of organisms and the way that landscape configuration affects them, beyond simplified indices of landscape structural attributes such as proximity and shape complexity. One novel approach has been to model landscapes according to graph theory, depicting habitats and corridors as networks of nodes and links, and calculating measures of connectivity based on this framework (Saura and Torné 2009). Considering connectivity as flows in a network has greater ecological utility than examining habitat structure alone; however, the simplification of connectivity into a binary framework remains limited in its ability to consider important ecological dimensions (Moilanen 2011). A binary landscape model is particularly limiting in urban settings where different land covers may represent varying levels of suitability and permeability, rather than absolute barriers and facilitators to wildlife flows (Tremblay and St. Clair 2011; Braaker et al. 2014). Instead, different land types may be used by a given species for different purposes or to different degrees (Mörtberg 2001). Approaches exist to move graph theoretic approaches beyond this binary limitation, such as the coupling of graph theory with least-cost path analysis using a landscape resistance map (e.g. Rayfield et al. 2010). However, current graph theoretical approaches can also become computationally unfeasible when dealing with high-resolution GIS grids (Moilanen 2011), which may be necessary to capture adequate levels of detail for urban ecological studies (Grafius et al. 2016). Additionally, they remain limited in their ability to consider the spatial patterns of the landscape between habitat nodes.

A relatively new approach for modelling functional connectivity in landscapes is to apply principles borrowed from electrical circuit theory: habitat patches and features in the landscape can thus be considered not only in terms of whether two patches share a connection, but also considering the resistance to that connection as a function of intervening land cover types, distances, and corridor traversibility (McRae et al. 2008). Unlike most previous methods, circuit theory operates on continuous map layers, thus considering multiple alternative connectivity pathways. This is believed to reflect ecological reality more accurately than either graph theoretical approaches or least-cost path analysis, both of which tend to focus solely on a single optimal path (McRae et al. 2008; Moilanen 2011). This approach has been successfully used in landscape genetic studies (e.g. Koen et al. 2012; Braaker et al. 2017) and, more recently, to model wildlife movement across landscapes (e.g. Koen et al. 2014; Jackson et al. 2016; McClure et al. 2016). The ability to deal in resistance rather than a binary habitat/non-habitat framework, coupled with the consideration of multiple pathways, may render circuit theory particularly valuable in urban habitats, where resident species are more likely to be adapted to stress, disturbance, and a complex habitat mosaic, and therefore be less reluctant to travel across anthropogenic surfaces when moving between habitat patches (McDonnell and Hahs 2015; Zhou et al. 2016). Nevertheless, urban wildlife may require a degree of habitat connectivity from urban features such as trees, parks, and rivers in order to survive or to thrive. Thus far, circuit theory has not been widely used in urban connectivity studies, however, despite possessing these notable advantages (cf. Bennie et al. 2014).

Regardless of the approach taken, studies of landscape connectivity have the greatest utility when they are based on empirical data relating to functional connectivity in that landscape (LaPoint et al. 2015; Shimazaki et al. 2016), or can have their results validated by comparison to empirical observations (e.g. Koen et al. 2014; Jackson et al. 2016; McClure et al. 2016). Unfortunately, such studies remain rare due to the difficulty and expense involved in acquiring direct measures relating to functional connectivity (Kindlmann and Burel 2008). Further, even when such data are available, the complexity inherent in ecological systems and interactions often limits the degree of observed variance that ecological models are capable of explaining (Møller and Jennions 2002).

In this paper, we model the habitat structure of a diverse urban environment using a circuit theory approach, with an emphasis on the importance of urban landscape structure to the movement of urban great tits (*Parus major*) and blue tits (*Cyanistes caeruleus*). The objective of this research was to use a circuit theory modelling approach to advance the understanding of how urban form influences patterns of movement among urban birds, and what landscape features and configurations appear to facilitate or impede urban wildlife connectivity. We then compare model results to empirical observations of bird abundance to explore the ability of the approach to explain observed ecological variability. Furthering our understanding of relationships between urban landscape form and ecological function can inform urban planning and design, allowing the creation of more ecologically connected cities, i.e. determining what goes where in a new urban area, or how we might retrofit features to improve connectivity. This in turn can improve not only the ecological health of cities, but also the quality of life for human urban residents through the provision of bird interactions and other encounters with nature (e.g. Cox et al. 2017).

## Methods

### Study area

**Fig. 1:**
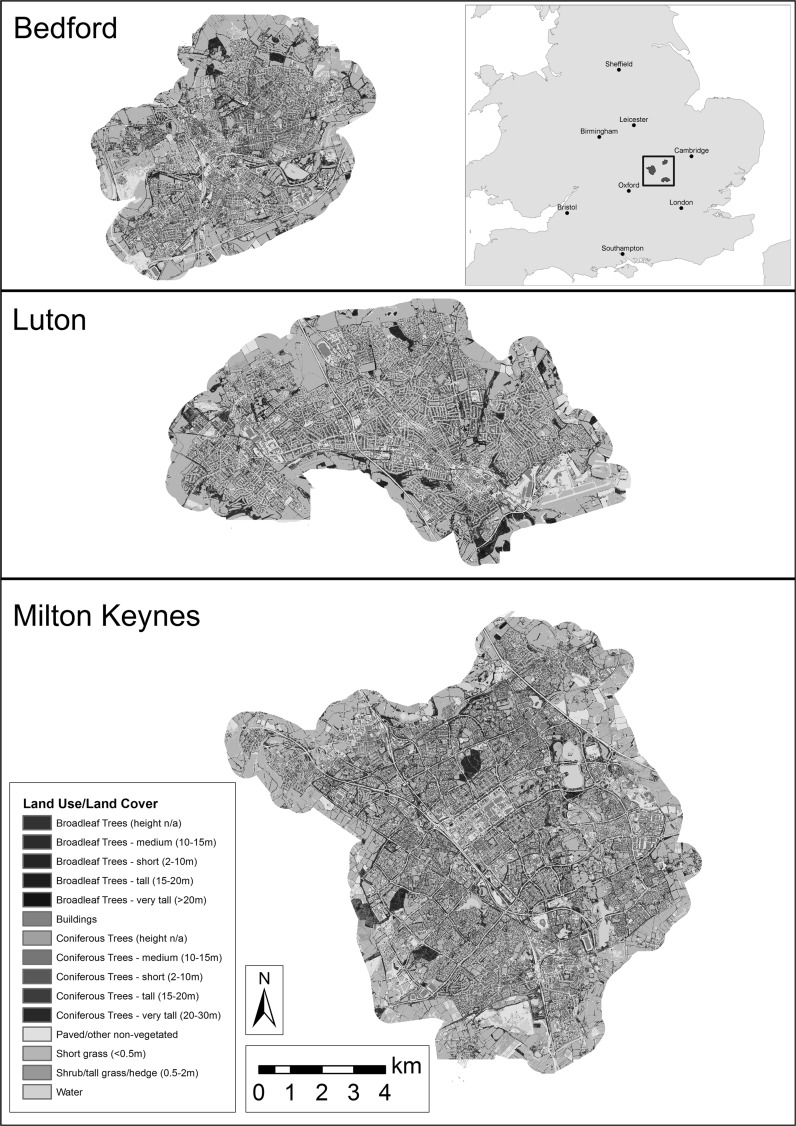
Study area showing locations and land use/land cover classification of Bedford, Luton, and Milton Keynes, UK

This project’s study area was the combined urban area of three large towns: Milton Keynes, Bedford, and Luton, UK (Fig. 1). Together the towns exhibit a broad range of urban forms and histories, representing much of the diversity found across the UK’s urban landscapes.

Milton Keynes is a planned ‘new town’ developed during the 1960s (52°0′N, 0°47′W), noteworthy for its unique road layout and urban form. Unlike the radial road network common to many UK urban areas, the town is structured around a grid of major roads designed for speed and ease of automotive travel (Peiser and Chang 1999). Milton Keynes is also characterised by a high coverage of public green space, possessing many parks and wooded foot and cycle paths (Milton Keynes Council 2015). The urban area possessed a population of 229,941 in 2011, covering an area of 89 km^2^ with a population density of 2584 inhabitants km^−2^ (Office for National Statistics 2013).

Bedford (52°8′N, 0°27′W) originated in the Middle Ages as a market centre, differing from Milton Keynes by possessing both a much longer history and a road network radiating outwards from its centre, like many British towns. Its 2011 population was 106,940 and the town covers 36 km^2^, with a population density of 2971 inhabitants km^−2^ (Office for National Statistics 2013).

Luton (51°52′N, 0°25′W) possesses an industrial heritage and saw much of its development during the nineteenth century. As such, its urban pattern largely consists of large industrial parks and residential ‘terraced’ housing. Here considered as the combined Luton/Dunstable urban area, the region had a 2011 population of 258,018 and covers 58 km^2^, with a population density of 4448 inhabitants km^−2^ (Office for National Statistics 2013).

A land use/land cover (LULC) map at 5 m resolution (each pixel representing a 5 × 5 m square) was used as the basis for much of the modelling and GIS analysis. This map was created from colour infrared aerial photography obtained from LandMap Spatial Discovery (http://landmap.mimas.ac.uk/). The imagery was taken on 2 June 2009 for Bedford, 30 June 2009 and 24 April 2010 for Luton, and 8 and 15 June 2007 and 2 June 2009 for Milton Keynes, based on cloud-free image availability. Vegetated and paved surfaces were separated according to a Normalised Difference Vegetation Index (NDVI) threshold. UK Ordnance Survey MasterMap data were subsequently used to identify buildings, water features, and major roadways. Subsequently, airborne LiDAR (Grafius et al. 2016) was used to categorize vegetation into height classes for short grass (<0.5 m), tall grass and shrubs (0.5–2 m), short trees (2–10 m), medium trees (10–15 m), and tall trees (>15 m).

### Urban bird flows

Great tits (*P. major*) and blue tits (*C. caeruleus*) were selected as the focal species for this research for several reasons: (1) both species represent typical UK woodland songbirds that have adapted to life in suburban environments, but nonetheless face breeding and foraging pressures from urbanisation (Mackenzie et al. 2014); (2) bird flow experiments focusing on urban feeder visitation in the same study area (Cox et al. 2016) found these species to be distributed evenly and widely across the area and different LULC types, thus confirming the presence of these species across the landscape and limiting geographic bias; (3) both are charismatic species to which urban residents respond favourably (Cox and Gaston 2015), thus providing a cultural ecosystem service in addition to their ecological roles. Additionally, *P. major* is thought to act as an indicator species for balanced urban food webs due to its sensitivity to green space connectivity (Hashimoto et al. 2005). The focus of this research was to balance functional connectivity of the target species with a broader landscape perspective, treating the target species as indicators of the urban landscape’s ability to facilitate wildlife movement, rather than attempting to represent all possible movement flows across a broad range of bird species. As such, individual types of movement (e.g. feeder visits, natal dispersal, etc.) and temporal scales (e.g. day-to-day behaviours, long-term gene flow) are not explicitly addressed, and instead considered in aggregate as determined by landscape structure.

Studies of bird movement in urban settings have highlighted the importance of landscape pattern to birds. Larger habitats with fewer gaps will increase foraging efficiency and decrease breeding costs in both *P. major* and *C. caeruleus* (Hashimoto et al. 2005; Hinsley et al. 2008), and *P. major* individuals prefer large woodland patches in their movements (Song and Kim 2015). However, in the absence of large woodlands, both species will readily make use of smaller vegetated patches as stepping stones (Hong et al. 2013). Given the often-fragmented and dispersed nature of urban green space, this characteristic meshes effectively with the continuous approach (as opposed to a binary habitat model) used by circuit theory modelling. In a study of the willingness of urban songbirds to cross non-habitat areas in Alberta, Canada, Tremblay and St. Clair (2009) found that 50% of birds were reluctant to cross gaps wider than 45 m. Birds also exhibited heighted reluctance to cross roads with heavy vehicle traffic, and water bodies were found to present a stronger barrier to movement than roads (Tremblay and St. Clair 2009). This study used chickadees (*Poecile atricapillus*) and warblers (*Dendroica petechia*), which are closely related and/or fulfil similar ecological functions to the species considered here. Related research found that avian travel between patches can be slowed considerably by the presence of multiple habitat gaps between the source and destination, while wooded corridors along barriers such as streams and roads can minimise these gaps and increase landscape connectivity (Tremblay and St. Clair 2011). Many of these findings are echoed by Shimazaki et al. (2016), who observed various forest bird species exhibiting high movement in woodland and lower movement in open land; buildings and water conversely were only considered to be intermediate barriers.

### Connectivity and circuit theory

Circuit-based connectivity was calculated using Circuitscape 4.0 (McRae et al. 2013). Using circuit theory to conceptualise landscape connectivity depends on an underlying resistance map, where each cell in the landscape is coded according to its relative unsuitability for use by the target species (i.e. more suitable habitat is assigned a lower resistance value and vice versa). Graph theory and electrical circuit theory are then coupled, using the resistance map, to produce a map of ‘cumulative current’ across the landscape as though it were an electrical circuit. Graph theory handles the treatment of pixels between habitat patches as nodes in a network, whereas current in this instance represents wildlife flows (Braaker et al. 2014). The resistance map can be thought of as effectively the inverse of a habitat suitability map, but with a focus on the willingness of individuals to move across a given cell (McRae et al. 2008). Resistance maps are generated by the user, often from LULC maps, with landscape classes and features interpreted according to their traversibility or effectiveness as a barrier to movement by the target species (Beaujean 2015). Such maps are most effective when considering characteristics that can be supported empirically as important to the target species, such as willingness to cross habitat gaps of different sizes and avoidance of certain landscape features. As such, the creation of an appropriate resistance map represents a major challenge of the circuit theory approach, being necessarily specific to a given study but also capable of encapsulating empirical findings and expert knowledge.

The circuit model runs on the resistance map, using random walk functionality to calculate the total resistance, and its opposite ‘current’, between pairs of user-defined ‘nodes’ representing core habitats that make up the sources and destinations for wildlife flows in the landscape. The specifics of where these core habitat nodes are located (representing sources and destinations for modelled flows and exhibiting a resistance value of zero) are determined by the user in accordance with the purpose and nature of the study. When each pairwise result is summed together, the resulting cumulative current map expresses a representation of the intensity of wildlife flow at each pixel. Pinch points and other important movement corridors can be easily identified from locations on the map with high values and narrow widths, thus identifying features and regions important to the connectivity of a study area (McRae et al. 2008). This in turn can be used to target conservation efforts and to highlight important relationships between landscape structure and ecological flows.

### Landscape resistance parameterisation

Based on the published findings described in the “Urban bird flows” section, the resistance parameters used in this study were selected with the intention of capturing a generalised picture of urban landscape connectivity using the described species as indicators. Habitat suitability and land cover resistance were conceptualised in an overall sense to model the connectedness of the urban environment for the various types and distances of dispersal that can be expected of *P. major* and *C. caeruleus*; the perspective taken was that of the landscape and population rather than the individual, so individual dispersal distances were not treated as a limiting factor. Both species were modelled together, as they are sufficiently similar in habitat preference, habit and behaviour that the same resistance parameters and core habitat locations should be appropriate for both.

Resistance values were assigned to mapped pixels based, first, on LULC class and, subsequently, modified by additional relevant factors and features (Table 1). Highly suitable woodland patches within the study area were assigned a low resistance value of 1 if they were larger than 5 ha in size and 2 if they were smaller than this but consisted of tall trees (>15 m), as these represent relatively ideal habitats, being old and structurally complex and theorised to be less impacted by edge effects and human use than smaller patches. Woodlands and shrublands outside of core habitat patches were assigned slightly higher resistance values according to their height to model their use as favourable to connectivity but with a small movement cost relative to the most suitable core habitats. Low grassland was parameterised not to count as favourable habitat but, being vegetated and thus less subject to human disturbance, to act as a weaker barrier to movement than sealed surfaces. Paved surfaces were parameterised as less suitable given their lack of vegetation, and water was selected as more extremely unsuitable, given Tremblay and St. Clair’s (2009) findings of the intense reluctance of woodland birds to cross it. Lastly, buildings were the least suitable base LULC class given both their lack of habitat amenities and their presence as physical barriers to movement in many cases. Modifiers to the above base values were then applied. Pixels greater than 45 m from woodland had 50 added to their initial resistance value after Tremblay and St. Clair’s (2009) findings of woodland birds being reluctant to cross gaps wider than this (implemented as a modifying effect, so the intervening land cover still plays a role; for example, a wide gap over short grassland will have a resistance value of 25 + 50 = 75, while a wide gap over water will have a resistance value of 45 + 50 = 95). Additionally, major road features (A roads, primary roads and motorways according to Ordnance Survey MasterMap data) had 20 added to their value in accordance with Tremblay and St. Clair’s (2009) documented aversion of woodland birds to busy road noise.

**Table 1:** Assigned resistance values (unitless, but on a 0–100 scale) by mapped land use/land cover (LULC) class, and modifications based on additional factors and features, for *P. major* and *C. caeruleus*

Class/feature	Assigned resistance value	Justification
Woodland patches larger than 5 ha	1	Song and Kim (2015) found *P. major* individuals prefer large woodland patches
Tall/mature woodland patches (>15 m)	2	Optimal habitat type (Perrins 1979); presence of tall trees indicates older, more structurally complex patches
All other woodland	5	Core habitat type but fewer ecological resources than mature stands
Tall grassland/shrub	10	Cover and some ecological resources
Short grassland	25	Some ecological resources but lack of cover
Paved/non-vegetated ground	30	No physical impediment to flight but few ecological resources
Water	45	Tremblay and St. Clair (2009) observed reluctance to cross waterbodies
Buildings	50	Physical impediment to flight
Land greater than 45 m from nearest woodland patch	Initial +50	Tremblay and St. Clair (2009) observed increased reluctance to cross gaps larger than 45 m
Major road (A roads, primary roads and motorways in OS MasterMap)	Initial +20	Tremblay and St. Clair (2009) observed reluctance to cross roads with heavy vehicle traffic

Selection of core habitat nodes used a combination of predicted habitat suitability based on patch size, contiguity, structure and landscape location, as well as empirical data in the form of observed abundances of the species of interest (described below in “Model evaluation and empirical abundance data” section). All woodland patches (combining broadleaf and coniferous, but woodlands in the study area are almost entirely broadleaf-dominated) greater than 10 ha were initially included, after which some were excluded on the basis of irregular shape (suspected to be dominated by edge effects and therefore not representative of high-quality core habitat), low observed bird abundance or locational redundancy with other nodes. Other patches were included based on nearby high observed abundance or perceived importance in the landscape’s connectivity network not already represented by other patches. Core nodes were primarily selected for their distribution around the perimeter of the urban areas in order to reduce bias introduced by node locations and to ensure an even coverage of the study area (cf. Koen et al. 2014). Exceptionally large and highly suitable habitats interior to the urban landscape were also included. The primary focus of this research was on wildlife behaviour within and across a mixed and complex urban environment, so core habitat nodes were placed to facilitate modelled movement across as much of the urban landscape as possible.

### Model evaluation and empirical abundance data

**Fig. 2:**
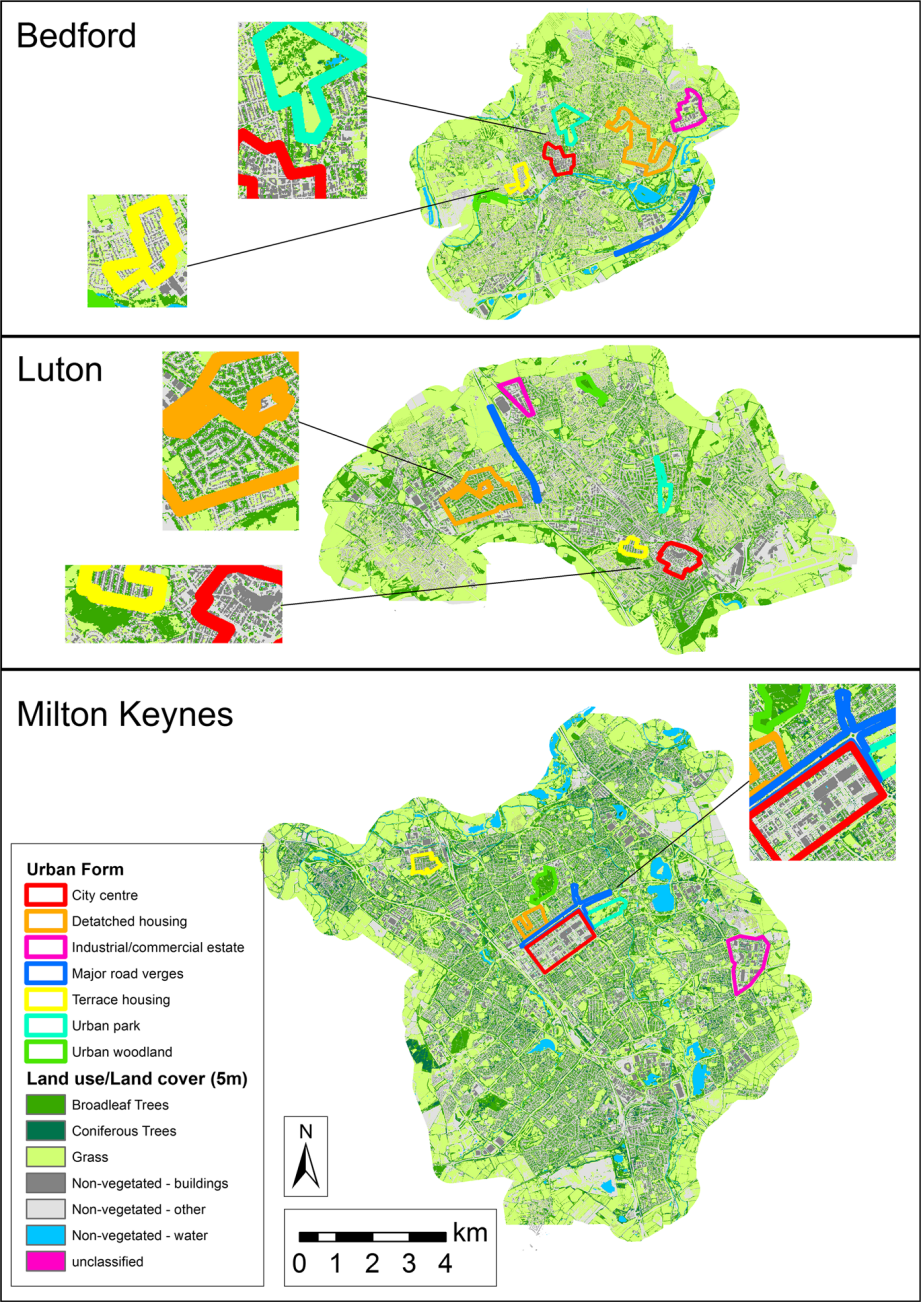
Study area showing samples of known urban form types

To investigate potential relationships between modelled connectivity and landscape structure, seven types of urban form common to UK cities were defined: city centres (i.e. central business districts), industrial estates, terraced (i.e. row/townhouse) housing, detached housing, major road verges, urban parks and urban woodland. For each type, one sample area believed to be representative of that type’s landscape structure was selected from each town (Fig. 2). The mean and standard deviation of connectivity model results within each urban form sample were then calculated in order to compare relative differences in modelled cumulative current between urban forms. Due to necessary co-location between core habitat nodes and suitable examples of urban woodland, a circular relationship between parameterised resistance and modelled current could not be avoided, so relatively high current values were anticipated for these areas. For other urban forms, the form represents an assemblage of different cover types and spatial patterns, and thus resistance values, according to the human use that defines them.

For the purposes of evaluating model performance, it was theorised that observed bird abundance would in principle be related to landscape connectivity such that it could act as a rough proxy against which current outputs could be tested. Circuitscape output has previously been tested against observed occurrence data on target species where actual abundance counts were not available (Koen et al. 2014; Jackson et al. 2016). In these cases, modelled current at recorded occurrence points was compared against current at random points; when the distribution of current values at occurrence points was found to be significantly higher than at random points, the model was considered successful. In this way, spatially explicit binary occurrence data on a target species can be used to evaluate Circuitscape performance. As studied here, the availability of count abundance data enabled a rare, more detailed comparison and an ability to test for correlation not offered by presence/absence data. Observed bird abundance and modelled connectivity current were suspected to share a positive but imperfect relationship, both being ecologically linked to habitat use by birds but not directly comparable to one another. Optimally, this approach would make use of empirical data on actual flows of wildlife across the landscape, but such data are particularly difficult, expensive and time-consuming to gather, and unfeasible at a city-wide scale and across an adequate diversity of land covers and urban forms (for an example of a neighbourhood-scale study, see Cox et al. 2016). This, in conjunction with the complexity of ecological systems and its documented impact on model uncertainty (Møller and Jennions 2002), meant that a relatively low degree of model fit was anticipated. The primary goal of the approach was to explore the utility of the modelling approach as a tool for understanding landscape form/function interactions and for explaining variation in observed abundance, with any positive relationship or explanation of variance considered to be informative, particularly from a type of data not commonly available for such studies.

Bird abundance was estimated using point count surveys conducted across the survey area during the summers of 2013 and 2014. Although these surveys were not directly concurrent with the collection of aerial imagery for land cover map creation described in “Study Area *”* (2009–2010), key land cover features and broad characteristics of bird abundance were believed to be appropriately consistent between these time periods given the scale of inquiry. Detailed behavioural and seasonal differences in bird movements were also unaccounted for by the nature and timing of observations, but were outside the scope of this research which remained focused on broad, landscape-level relationships involving breeding bird populations. Observation points (n = 454) were positioned within 1,16,500 m × 500 m grid squares that had been randomly selected using a stratified sampling design to account for variation in urban form. Each grid square contained up to four points, located at least 200 m apart and 100 m from the square boundary. All points were surveyed twice each year, once in May and once in June and at times when birds are most detectable (0600–1000 h), in order to estimate overall, relative, abundance as closely as possible. Each point count consisted of a 10 min observation, divided into 2-min blocks, during which trained observers recorded all birds seen and heard within five distance categories (0–20, 20–40, 40–60, 60–100, and 100–200 m). Count data for *P. major* and *C. caeruleus* were restricted only to include individuals recorded at distances of ≤60 m from the observer, as this was considered to be the approximate detection limit for these species within an urban context; this was supported by a sharp decline of observations in distance bands >60 m, and will have reduced variation in detectability between different land cover types caused by the ease of observation. Abundance estimates were calculated at each observation point by summing the maximum counts of singing and non-singing *P. major* and *C. caeruleus* individuals in each distance band up to 60 m, taking the maximum value across all point visits in a given year as an indicator of that year’s abundance, and then calculating the mean of this across the two years of observation (2013 and 2014). This produced an estimate of overall abundance for the species of interest at each point, which could then be compared to the circuit model results at those points.

Additionally, abundance data for great spotted woodpecker (*Dendrocopos major*) and chiffchaff (*Phylloscopus collybita*) were compared to the cumulative current map (Table 2). These species are woodland birds that have broadly similar habitat dependencies to blue and great tits in terms of their reliance on habitats with mature trees (Mason 2001 for *P. collybita* depending on woodland interiors; Gil-Tena et al. 2013 for *D. major* depending on mature woodlands, dead wood, and high habitat connectivity), but with more woodland-specialist tendencies than *P. major* and *C. caeruleus*. Model parameters were not specifically devised for these species, so this comparison represented a way in which the ‘breadth’ of the model’s suitability for a wider species pool could be evaluated.

**Table 2:** Summary statistics for bird species abundance observations by sampling point, averaged over observation years (2013 and 2014)

	Blue tits (*Parus major*)	Great tits (*Cyanistes caeruleus*)	Chiffchaffs (*Phylloscopus collybita*)	Great spotted woodpeckers (*Dendrocopos major*)
Total	887	414	68	60
Mean	2.11	1.60	1.07	1.13
SD	1.06	0.93	0.46	0.53
Minimum	1	1	1	1
Maximum	8	8	2	3
*N*	420	259	62	53

Since the values of the cumulative current maps are highly influenced by the number of node pairs, and each town contained a different number of core habitats (Bedford 6; Luton 12; Milton Keynes 13), the maps exhibited different data ranges between towns and were rescaled to values between zero and one to facilitate valid relative comparisons between urban areas. Comparison with observed abundance data was carried out by defining a 60 m radius around each observation point to represent the effective area of the sample, and calculating the mean rescaled current within this area resulting from circuit analysis. Sampled mean current values were then compared to observed abundance of target bird species using a generalised linear regression model to test model fit.

## Results

### Spatial patterns

**Fig. 3:**
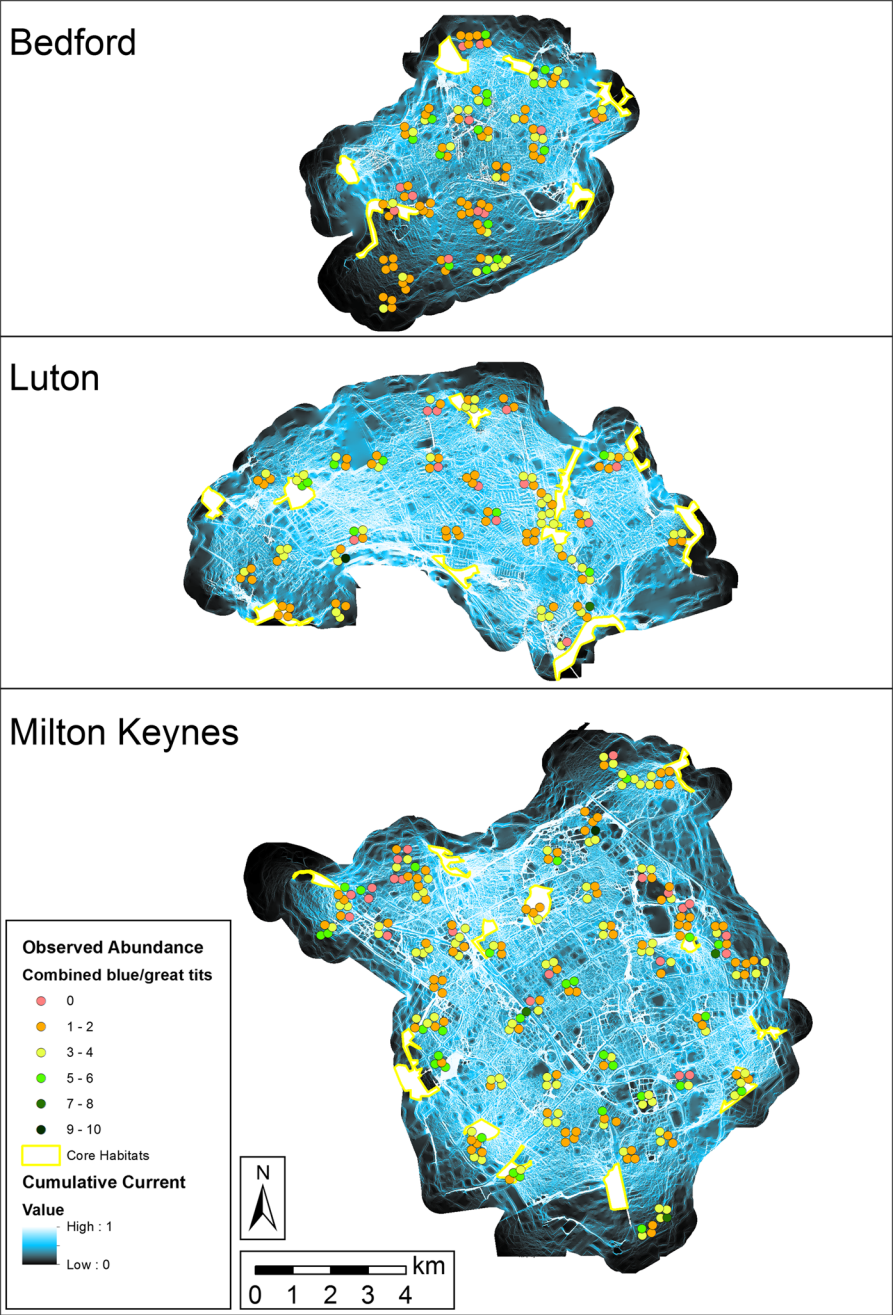
Modelled cumulative current (rescaled to facilitate comparison between towns and displayed by histogram equalisation to show landscape patterns due to relatively very high values at node locations) and observed abundance values (mean of 2013–2014) for combined blue tits (*C. caeruleus*) and great tits (*P. major*) in Bedford, Luton, and Milton Keynes, UK. Core habitat node locations are also shown

The modelling of landscape connectivity in Circuitscape for *P. major* and *C. caeruleus* resulted in maps of cumulative ‘current’ for each town. The intensity of current here is used as a proxy for wildlife movement at each pixel on the landscape and was calculated between each pair of core habitats in each town. The results of each pair were then summed to form a cumulative current map which expresses the intensity of potential flow at each pixel when all core habitat pairs are considered (Fig. 3).

Cumulative current across Bedford was visibly affected by the distribution of core habitat areas, with the southwestern region of the town exhibiting decreased modelled flow values due to the presence of only one major habitat patch in that region. The proximity of core habitats to one another in other regions of the town appears to generate increased current in the areas surrounding and joining them. Outside of these habitats, modelled flows can be seen to follow wooded corridors where available, while in other cases moving between small wooded islands and dispersing between them.

Modelled movement patterns in Luton/Dunstable appeared to be similar, but with a greater visual emphasis on loosely-networked corridors of wooded and mixed habitats, such as through residential gardens between rows of terraced housing. Vegetated corridors along major transport arteries such as the railway and the M1 motorway also stand out as important to modelled bird flows.

In Milton Keynes, the effect of vegetated road verges is the most visually striking pattern, with the major grid road network easily discernible in the current map. This suggests that the grid road verges may serve as important wildlife corridors within the urban environment. Modelled current flows along wooded corridors in residential districts and linear parks also suggest the importance of these features, especially in contrast to the city centre and industrial estates, where they are present but more restricted to a sparser flow network.

### Quantitative comparisons

In the initial cumulative current maps (not shown, but different from Fig. 3 only in data scale), Bedford contained notably lower overall modelled current values (maximum 5.13, mean 0.05) than Luton (maximum 11.89, mean 0.21) and Milton Keynes (maximum 12.57, mean 0.11) due to its possession of fewer core habitat nodes. While the number of core habitats has a clear impact on the connectivity of a landscape, the rescaled values were the focus of numerical comparisons in order better to consider the character of the study area’s landscapes with respect to connectivity.

After rescaling, Luton possessed the highest mean connectivity (0.0175; averaged across all pixels contained in that town) but also the highest variability (SD 0.1211). Milton Keynes (mean 0.0087, SD 0.0856) and Bedford (mean 0.0098, SD 0.0933) possessed lower values. Each town is made up of different spatial combinations of urban forms and landscape patterns that contribute to these numerical differences and confound direct comparisons, so it was suspected that a comparison of mean current values between samples of known urban form types in the landscape would prove more informative. These urban forms represent human use-based assemblages of the land cover types that resistance values were based on with particular spatial patterns, rather than those land covers themselves (Table 3). Consistent with expectations, city centre and industrial estate samples demonstrated the lowest current values and urban woodland the highest, although woodlands were anticipated to exhibit particularly high current values due to their co-location with core habitat nodes which introduced circularity in current calculation. Industrial estate and terraced housing samples shared nearly the same mean current value, but terraced housing exhibited greater variability. Sampled green corridors along major road verges demonstrated relatively high connectivity values. However, all forms exhibited sufficiently high variability that no statistically significant differences were present between different urban forms apart from urban woodlands, which were higher than all others.

**Table 3:** Mean and standard deviation of rescaled current values in sampled locations of known urban forms and total study area

Urban form	Mean relative current	SD
City centre	0.0021	0.0011
Industrial estate	0.0024	0.0015
Terrace housing	0.0024	0.0019
Detached housing	0.0039	0.0020
Urban park	0.0044	0.0041
Major road verges	0.0238	0.1412
Urban woodland	0.8238	0.3282
Total study area	0.0112	0.0976

### Comparison with observed bird abundance data

The results of the Circuitscape model, taken as the mean current value in a 60 m radius around each observation point, were tested against observed abundance data for *P. major* and *C. caeruleus* combined, using a generalised linear regression model with a standard least squares estimation method (α = 0.05). Relationships were also tested against all target species individually. Due to consistent skew in the data, modelled current and all observed abundance data were log transformed for analysis.

As tested and parameterised here, modelled current exhibited too weak a relationship with combined blue tit and great tit abundance to show a significant relationship (R^2^ = 0.015, p = 0.0657). When tested against individual species, blue tits showed a weak but positive significant relationship (R^2^ = 0.012, p = 0.0296) whereas great tits did not (R^2^ = 0.015, p = 0.0657). Unsurprisingly, the secondary species showed poorer fit and no significant relationship (R^2^ = 0.011 and p = 0.4569 for *P. collybita*; R^2^ = 0.031 and p = 02742 for *D. major*).

## Discussion and conclusions

Our primary objective in conducting this research was to explore, through a circuit theory modelling framework, how the abundance of two urban-adapted woodland bird species is affected by variation in patterns of movement due to urban form and landscape pattern. Such effects are likely to reflect the birds’ perceptions of habitat quality due to the ease of movement between physically separated patches providing complementary resources or a single, functionally larger, area, or to reflect population dynamics via the ease of annual dispersive movements into high-quality patches (e.g. Dunning et al. 1992; Fahrig et al. 2011). We have investigated this using a broad, landscape-scale perspective that avoids the need for direct data on bird flows, instead making use of observed abundance data as a proxy and hypothetical covariate with bird movement across different urban forms. These abundance data, although not directly analogous to wildlife flow, nevertheless represent an improvement over commonly-used binary presence/absence data, because counts are likely to be more sensitive to environmental variation than presence.

In all modelled cases, the occurrence of trees appeared to act as a primary driver of spatial patterns of connectivity. Although the importance of large contiguous woodland was assumed a priori for model parameterisation, trees occurring elsewhere on the landscape as individuals or in small stands appeared to act as islands enabling modelled movement of woodland species across large expanses of non-habitat. Intermediate-sized patches of tree cover tended to display high current values relative to surrounding areas and the appearance of forming networks of partial corridors, facilitating modelled movement between larger habitats.

The spatial results of the analysis strongly suggest that wooded verges to major roads, railways and streams play a major role in facilitating connectivity in urban landscapes. High-current networks are apparent in the major grid roads of Milton Keynes, the M1 motorway and railway in Luton, and the River Great Ouse in Bedford. Linear parks following stream corridors in Milton Keynes were also apparent as high-current areas, and residential housing estates in Bedford containing patches of tall trees with conjoined canopies appeared to act as high-current transport ‘junctions’ for modelled flows between larger core habitats. These high-current areas potentially represent ‘pinch points’ where flow is high but connectivity may also be most vulnerable. The loss of habitat in such areas could thus prove to be the most damaging to the connectivity of the urban environment for the species of interest, depending on how much redundancy is present. The removal of high-current corridors would have a reduced impact where other nearby corridors can provide similar connections, but the loss of more isolated corridors could have a more significant negative impact on the connectivity of the landscape by isolating core habitats from one another. Individually, areas of trees on the landscape possessing high current values is unsurprising given the way in which the resistance map and core node locations were parameterised; however, the emergence of high-current networks linked to key features on the urban landscape outside of the core habitats is of potential relevance to urban planners and ecologists.

The connectivity of a landscape is driven by the spatial patterns and landscape structure present in an area, but the underlying land covers and their relative occurrence act as a foundation for these patterns and structures. Radford et al. (2005) found that landscapes where effective habitat cover comprises less than 10% of the environment become ecologically pressured such that they will probably experience rapid declines in bird species richness. Concurrently, habitat modelling for *P. major* in Osaka suggested that 10% tree cover for areas outside large parks was realistically necessary to create an ecologically connected and sustainable city (Hashimoto et al. 2005). Within the study area here, Milton Keynes, Luton/Dunstable and Bedford possess proportional tree covers of 25, 22, and 16%, respectively, all reasonably above this recommendation (total combined study area land cover proportions were 24% broadleaf trees, 2% coniferous trees, 34% short grass, 7% shrub/tall grass/hedge, 8% buildings, 23% paved/other non-vegetated, and 2% water). The relative degrees to which each town exceeds the 10% threshold loosely match their initial maximum cumulative current values, supporting the theory of a positive relationship between proportional habitat cover and overall connectivity of the urban landscape. As modelled here, the initial numerical current results are primarily driven by the number of habitat nodes in each town rather than the intervening landscape configuration of that town; nevertheless the occurrence of large, core habitats is an important contributor to connectivity and biodiversity in urban environments (Fernández-Juricic and Jokimäki 2001). The rescaled values enabled a more appropriate comparison between the three towns; after rescaling, Luton exhibited a considerably higher mean current value than Milton Keynes or Bedford. Although these differences are expected to be driven by many interrelated factors, the relative tree covers, and thus available woodland habitat, will play a key role in the relative connectivity of each town. The greater presence of water bodies in Milton Keynes and Bedford may also influence this result. Mean current results are influenced positively by the presence of patterns and forms favourable to connectivity, and negatively by patterns and forms detrimental to connectivity; here, the relatively high values in Luton are thus an effect of its high tree cover coupled with its lower water cover (water features possess numerous ecological benefits, but in this context are detrimental to woodland bird movement). As such, this result represents a strength of the circuit theory modelling approach for its ability to consider the impacts of different land covers and structural patterns in combination, delivering findings that might not be reached otherwise.

The comparison of current values among the different urban forms did not demonstrate statistically significant differences between urban forms; the only exception to this was urban woodland, which was anticipated to exhibit higher values than other forms due to its co-location with core habitats, thus introducing a circular relationship in current calculations. The values suggest that in our study area, industrial estates and terraced housing may exhibit similar levels of connectivity to woodland birds, but with terraced housing subject to greater variability. The spatial patterns involved with this form of row housing can potentially provide both linear corridors and barriers to woodland bird movement, depending on orientation and tree cover. City centre and industrial estate samples were expected to exhibit relatively low current values, as both forms can contain tree cover and small green corridors but are commonly typified by large expanses of impervious surfaces. Major road verges, by contrast, may act as valuable movement corridors (e.g. Tremblay and St. Clair 2011); however their high current variability suggests this may only be true in some cases or at specific points in the network. Additionally, the roads themselves act as barriers to movement, presumably leading to a complex mixture of conflicting effects. Across nearly all urban forms the variability in current values was high enough to preclude statistically significant differences between forms, suggesting that many forms, at least as defined here, may be too diverse in spatial structure to act as effective covariates for ecological function. Further, the relationships between urban form and wildlife movement patterns may also be less deterministic or more complex than is generally assumed.

Visually it was difficult to discern any clear spatial relationships between observed abundance counts and model output or underlying landscape structure. Modelled current values were highly skewed (as is normal for Circuitscape output), with core habitat nodes and some very high-traffic areas possessing values close to 1 but much of the landscape’s variability falling below 0.01. Evaluation was consistent with the ‘value at observed points’ method described by McClure et al. (2016), with the rescaled current values being averaged over a 60 m radius around the observation points in accordance with the range of recorded bird sightings. Previous research testing the outputs of Circuitscape modelling against wildlife observations (e.g. Koen et al. 2014; Jackson et al. 2016) has made use of occurrence at locations but rarely had access to abundance counts, and as such model fit is problematic to compare directly.

Combined blue tit and great tit abundance, despite being the conceptual driver of model parameterisation, did not show a significant relationship with modelled current. When individual species were considered, blue tit abundance did show a weak but positive and significant relationship; however great tits, chiffchaffs and great spotted woodpeckers did not. The results suggest a potential for circuit theory modelling approaches to explain a small degree of observed bird abundance, as it did here for *C. caeruleus,* but further refinement is clearly needed to appropriately model most individual species. The low explanatory power of modelled current for observed bird abundance is not surprising, because connectivity (representing potential wildlife flow) is just one of the probable drivers of variation in local tit abundance (representing areal counts of individuals), and the analysis was not intended to provide a complete explanation of landscape influences on bird abundance. Other likely drivers include vegetation type and structure (e.g. age and species of trees; K.E. Plummer & G.M. Siriwardena, unpublished), levels of disturbance from humans or cats (Bonnington et al. 2013), bird social structures (Farine and Sheldon 2016) and the presence of nestboxes (Davies et al. 2009). Such features determining core habitat resources and influencing breeding and nesting behaviour may be very small (e.g. individual trees or artificial nest boxes) and thus defy representation in a pixel-based analysis. Finally, there may be important differences between the elements of landscape structure and configuration that influence bird movement across the landscape and those that can be feasibly captured and mapped for use in models. All such factors are potential causes of noise around the relationship with connectivity. In general, ecological studies deal with many factors and a high degree of uncertainty, commonly resulting in low predictive power and relatively little of a system’s overall variance being explained, even when research is widely considered to be successful (Møller and Jennions 2002). However, that a positive relationship is present supports the assumptions and conceptual framework of the model approach, but also suggests room for refinement or further study.

Agreement between model results and observed abundance may have been further impacted by sampling error in the observational data. These data were gathered in a standardized manner; however, discrepancies may exist in the ease of sightings between different land covers and other factors, although the limiting of observations to 60 m was intended to minimize such problems, and there is no reason to suspect that such variations in detectability would cause bias in the assessment of effects of connectivity at scales greater than a 60 m radius.

This study considered local breeding abundance of tits, but these species are resident in the UK and they are generally more mobile in the winter, moving around the landscape in response to food availability (Ekman 1989; Wernham et al. 2002). Hence, winter abundance data may provide a more sensitive measure of connectivity effects and would provide a valuable future extension of this study. In addition, further empirical study on bird flow, converted to areal movement intensity values, would present the most valid comparator for circuit model results describing urban landscape forms with different levels of connectivity, but such measurements are costly and difficult to obtain even over small scales, requiring techniques such as capture-mark-recapture, or radio- or satellite-tracking. Such past studies (e.g. Tremblay and St. Clair 2009, 2011; Cox et al. 2016; Shimazaki et al. 2016) have produced invaluable information for understanding the ways in which birds move across urban landscapes, but remain rare due to the difficulties and feasibility of carrying out such research. Biases due to the relative ease of catching or following individuals in different habitats are likely to be a problem in such studies, while the logistics mean that sample sizes are likely to be small. Given that bird movement behaviour can be expected to vary according to characteristics of individuals, such as age and sex, there are further sources of noise in such direct measurements that do not apply to larger-scale analyses of abundance data. Future small-scale connectivity studies will benefit from greater research on how such individual behaviours affect movement patterns, whereas larger-scale studies will benefit from a more developed understanding of whether or not such behaviours scale up to exhibit significant impacts on landscape-scale movement.

The explanatory power of the current model for other bird species (*D. major* and *P. collybita*) was inadequate in both cases. Observations of both species were less frequent than those of the tit species, so statistical representation was weaker (total average observed abundance across both years for blue tits: 887; great tits 414; chiffchaffs 68; great spotted woodpeckers 60; see also Table 2). Although the model for blue tits was stronger, as the sample sizes would predict, the positive (if insignificant in this case) relationships for the other species suggest that the model may have explanatory value for other species such as *D. major* and *P. collybita* if refined and adapted. Abundance counts between *D. major* and *P. collybita* (not shown) were poorly correlated with one another, suggesting divergent behavioural and habitat characteristics, but supporting the general value of connectivity (as revealed by the current model) for the urban bird community. This suggests that circuit theory approaches can provide a valuable addition to the arsenal of data sources for modelling the habitat relationships and responses of birds to variation in urban form, and therefore for predicting the effects of development and urban management on biodiversity. However, our results suggest that numerous other factors besides connectivity introduce variability to urban bird abundance, and further research will continue to benefit from close collaboration between ecological modellers and avian ecologists.

The approach demonstrated here represents a way in which circuit theory can be used to assess the ecological connectivity of urban environments. By changing the manner in which core habitats are selected and the criteria for determining resistance values of the underlying landscape, this approach could be adapted to study a wide variety of urban wildlife species and how the structure of the landscape facilitates or impedes their movement. The greatest challenge in most such situations remains the availability of empirical data to support the selection of appropriate parameters, followed by the uncertainty involved in the selection of appropriate resistance values and core habitats for a given species and landscape. As computational feasibility improves, future efforts could inform this research by comparatively testing results generated using different approaches to resistance surface parameterisation, thus seeking an optimal set of parameters for any given landscape and species. As knowledge grows regarding the behaviours of different species with respect to their use of the landscape, models such as these can be used with increasing accuracy and validity to predict the importance of spatial patterns and features to wildlife. This in turn can be used to deliver more accurate guidance to planners and decision-makers in designing well-connected, ecologically sustainable developments optimised to deliver ecosystem service benefits for all residents, irrespective of location, by offering a prescription as to where to invest in green infrastructure.
